# BMC Oral Health reviewer acknowledgement 2014

**DOI:** 10.1186/1472-6831-15-13

**Published:** 2015-02-04

**Authors:** Magdalena Morawska

**Affiliations:** BioMed Central, Floor 6, 236 Gray’s Inn Road, London, WC1X 8HB UK

## Abstract

The editors of *BMC Oral Health* would like to thank all of our reviewers who have contributed to the journal in Volume 14 (2014).

**Irene Aartman**

Netherlands

**Jenny Abanto**

Brazil

**Guy Adami**

USA

**Charlene Africa**

South Africa

**Pius Agbenorku**

Ghana

**Hermann Agis**

Austria

**Basaruddin Ahmad**

Malaysia

**Rahil Ahmadi**

Iran

**Jun Aida**

Japan

**Aliye Akcali**

Turkey

**Merve Akcay**

Turkey

**Reem Alansari**

Saudi Arabia

**Asmaa Alkhtib**

Qatar

**Abdullah Alswuailem**

Saudi Arabia

**Luana Alves**

Brazil

**Yulia Anopa**

UK

**Robert Anthonappa**

Australia

**Raquel Antoniazzi**

Brazil

**Heliton Spindola Antunes**

Brazil

**Jose Leopoldo Ferreira Antunes**

Brazil

**Daniel Apolinario**

Brazil

**Takafumi Arimoto**

Japan

**Luciana Armada Dias**

Brazil

**Jason Armfield**

Australia

**Wolfgang H. Arnold**

Germany

**Kristina Arnrup**

Sweden

**Peter Arrow**

Australia

**Rodrigo Arthur**

Brazil

**Paul Ashley**

UK

**Álfheiður Ástvaldsdóttir**

Sweden

**Sheyla Auad**

Brazil

**Avadhoot Avadhani**

India

**Paul Averley**

UK

**Lama Awawdeh**

Jordan

**Bjorn Axtelius**

Sweden

**Premalatha B R**

India

**James Bader**

USA

**Kwang-Hak Bae**

South Korea

**Zahra Baghani**

Iran

**Joanna Baginska**

Poland

**Ivona Bago Juric**

Croatia

**Hyoung-Seon Baik**

South Korea

**Azam Bakhshandeh**

Denmark

**Jeff Banas**

USA

**Asgeir Bårdsen**

Norway

**Mark Bartold**

Australia

**Ulku Baser**

Turkey

**Leonidas Batas**

Greece

**Paul Batchelor**

UK

**Asli Baysal**

Turkey

**David Bearn**

UK

**Sema Becerik**

Turkey

**Cristiane Bendo**

Brazil

**Vanessa Bernardes**

Brazil

**Bishal Bhandari**

UK

**Maria Biazevic**

Brazil

**Yvonne Blair**

UK

**Ali Borzabadi-Farahani**

UK

**Dieter Daniel Bosshardt**

Switzerland

**Peter Bottenberg**

Belgium

**Mariana Braga**

Brazil

**Marius Bredell**

Switzerland

**Susan Bridges**

China

**Jonathan Broadbent**

New Zealand

**Paul Brocklehurst**

UK

**Josef Bruers**

Netherlands

**Weber Bueno De Andrade**

Brazil

**Yvonne Buischi**

USA

**Caroline Bulsara**

Australia

**Michael Büttner**

Belgium

**Michele Callea**

Italy

**Guglielmo Campus**

Italy

**George Candeiro**

Brazil

**Ismail Davut Capar**

Turkey

**Vinicius Carrard**

Brazil

**Jorge Castillo**

Peru

**Juliano Cavagni**

Brazil

**Peter Celec**

Slovakia

**Mevlut Celikoglu**

Turkey

**Paulo Cesar**

Brazil

**Benjamin Chaffee**

USA

**Lin-Yang Chi**

Taiwan

**Chun-Pin Chiang**

Taiwan

**Ava Chow**

Canada

**Sergio Chrisopoulos**

Australia

**Lisa Christensen**

Denmark

**Bradley Christian**

Australia

**Chun-Hung Chu**

Hong Kong

**Sara Cioccari Oliveira**

Netherlands

**Jan Clarkson**

UK

**Juliana Clemente-Napimoga**

Brazil

**Leonard Cohen**

USA

**Ana Paula Colombo**

Brazil

**Georg Conrads**

Germany

**Rosalía Contreras-Bulnes**

Mexico

**Mabel Cordeiro**

Brazil

**Patrícia Corrêa-Faria**

Brazil

**Luciane Costa**

Brazil

**Yuri Costa**

Brazil

**Lisa Ann Coulthwaite**

UK

**Leonard Crocombe**

Australia

**Sofie Cronholm**

Sweden

**Donald Curtis**

USA

**Barbara Cvikl**

Austria

**Ewa Czochrowska**

Poland

**Cristiane Da Mata**

Ireland

**Dorothea Dagassan**

Switzerland

**Debora Dalpian**

Brazil

**Michele D'Attilio**

Italy

**Peter Day**

UK

**Cees De Baat**

Netherlands

**Andrea De Silva**

Australia

**Elsa Delgado**

UK

**Matthias Dettmer**

Switzerland

**Curtis Deutsch**

USA

**Kimon Divaris**

USA

**Virginia Dodd**

USA

**Manoela Domingues Martins**

Brazil

**Anna Dongari-Bagtzoglou**

USA

**Christof Dörfer**

Germany

**Bernadette Drummond**

New Zealand

**Marco Antonio Hungaro Duarte**

Brazil

**Carlos Eduardo Da Silveira Bueno**

Brazil

**Sigrun Eick**

Switzerland

**Daisuke Ekuni**

Japan

**Marlies Elfrink**

Netherlands

**Arjuna Ellepola**

Kuwait

**Gülnur Emingil**

Turkey

**Dan Ericson**

Sweden

**Rashidah Esa**

Malaysia

**Daniel Etienne**

France

**Vanessa Euzebio Alves**

Brazil

**Carla Evans**

USA

**Hani Fadel Saudi**

Arabia

**Ali Farahani**

UK

**Najat Farsi Saudi**

Arabia

**Olawunmi Fatusi**

Nigeria

**Stefano Fedele**

UK

**Fernanda Ferreira**

Brazil

**Joao Ferreira**

Singapore

**Sandra Fidel**

Brazil

**Carlos Marcelo Figueredo**

Brazil

**Marcellus Fischer**

Germany

**Ricardo Fischer**

Brazil

**Morenike Folayan**

Nigeria

**Michael Foley**

Australia

**Lyndie Foster Page**

New Zealand

**Helena Fransson**

Sweden

**Paulo Frazao**

Brazil

**Maria Do Carmo Freire**

Brazil

**Osamu Fujise**

Japan

**Jenny Gallagher**

UK

**Jose A. Garcia-Sanz**

Spain

**Roopadevi Garlapati**

India

**Istvan Gera**

Hungary

**Geoffrey Gerstner**

USA

**Barry Gibson**

UK

**Fiona Gilchrist**

UK

**Nikolaos Gkantidis**

Switzerland

**Nikolaos Gkranias**

UK

**Richard Glass**

USA

**Marilia Goettems**

Brazil

**Ali Golkari**

Iran

**Carolina Gomes**

Brazil

**Juliana Gomez**

UK

**Agnieszka Gornowicz**

Poland

**Mousumi Goswami**

India

**Daniela Goursand**

Brazil

**Gareth Griffiths**

UK

**Reinhard Gruber**

Switzerland

**Arndt Guentsch**

Germany

**Ekta Gupta**

UK

**Saurabh Gupta**

India

**Ulvi Gursoy**

Finland

**Kruthika Guttal**

India

**Ana Estela Haddad**

Brazil

**Sebastian Hahnel**

Germany

**Magnus Hakeberg**

Sweden

**Sema Hakki**

Turkey

**Demetrios Halazonetis**

Greece

**Jane Harford**

Australia

**Rebecca Harris**

UK

**Akiko Haruyama**

Japan

**Bassam Hassan**

Netherlands

**Songlin He**

China

**Lisa Heaton**

USA

**Mithra Hegde**

India

**Masahiro Heima**

USA

**Andreas Hellak**

Germany

**Ute Hempel**

Germany

**Bruno Herera**

Brazil

**Fernando Herkrath**

Brazil

**Christian Hirsch**

Germany

**Ha Hoang**

Australia

**Thomas Hoffmann**

Germany

**Ariane Hohoff**

Germany

**Akishige Hokugo**

USA

**Richard Holmes**

UK

**Christopher Holmgren**

France

**Catherine Hong**

Singapore

**Kiyonobu Honma**

USA

**Attila Horvath**

Hungary

**Xuwei Hou**

China

**Deyu Hu**

China

**John Hughes**

UK

**Timothy Paul Hyde**

UK

**Betül Ilhan**

Turkey

**Jose Imparato**

Brazil

**Marita Rohr Inglehart**

USA

**Javier Irurita Olivares**

Ecuador

**Kazuyuki Ishihara**

Japan

**Inyang Isong**

USA

**Kanade Ito**

Japan

**Jacques Izard**

USA

**Nick Jakubovics**

UK

**Philip James**

UK

**Claude Jaquiery**

Switzerland

**Juliana Jardim**

Brazil

**Lj Jin Hong**

Kong

**Colwyn Jones**

UK

**Kelly Jones**

Australia

**Birgitta Jönsson**

Norway

**Carina Källestål**

Sweden

**Khady Ka**

Canada

**Dieudonne Kaimbo Wa Kaimbo**

Democratic Republic of the Congo

**Anmol Kalha**

India

**Kivanc Kamburoglu**

Turkey

**Noriaki Kamio**

Japan

**Adriane Kamulegeya**

Uganda

**Ertugrul Karatas**

Turkey

**Ajay Kashi**

USA

**Mehmet Emin Kaval**

Turkey

**Yasuhiko Kawai**

Japan

**Yumiko Kawashita**

Japan

**Roger Keller Celeste**

Brazil

**Arthur Kemoli**

Kenya

**Robert Kerstein**

USA

**Zohaib Khan**

Germany

**Yuichiro Kikuchi**

Japan

**Jin-Bom Kim**

South Korea

**Haeyoung Kim**

South Korea

**Shiho Kino**

UK

**Shiho Kino**

Japan

**Mitsuo Kishi**

Japan

**Chiaki Kitamura**

Japan

**Thomas Klinke**

Germany

**Dimitrios Kloukos**

Switzerland

**Anne Koerber**

USA

**Klaus König**

Netherlands

**Jan Harm Koolstra**

Netherlands

**Sudaduang Krisdapong**

Thailand

**Sree Vidya Krishna Rao**

Australia

**Purnima Kumar**

USA

**Elena Labajo González**

Spain

**Satu Lahti**

Finland

**Gabriela De A. Lamarca**

Brazil

**Markku Larmas**

Finland

**Vladimir Lazarevic**

Switzerland

**Soraya Leal**

Brazil

**Sam Leary**

UK

**Gillian Lee**

Hong Kong

**Claudio Leles**

Brazil

**Erika Lencova**

Czech Republic

**Ryan Lennon**

USA

**Tathiane Lenzi**

Brazil

**Roos Leroy**

Belgium

**Duangjai Lexomboon**

Sweden

**Huan-Cai Lin**

China

**Helen Liversidge**

UK

**Edward Lo**

Hong Kong

**Thais Lopez**

Brazil

**Anna Louropoulou**

Netherlands

**Hai-Xia Lu**

China

**Heinz-Theo Lübbers**

Switzerland

**Hans Ulrich Luder**

Switzerland

**Richard Macey**

UK

**Arsalan Malik**

Pakistan

**Manuele Mancini**

Italy

**Leonardo Marchini**

USA

**Rodrigo Mariño**

Australia

**Luc A.M. Marks**

Belgium

**Márcia Marques**

Brazil

**Zoe Marshman**

UK

**Flavia Martinez De Carvalho**

Brazil

**Joyce Rose Masalu**

Tanzania

**Cinzia Maspero**

Italy

**Erin Masterson**

USA

**Manu Mathur**

India

**Yusuke Matsuyama**

Japan

**Juliana Mattos-Silveira**

Brazil

**Colman Mcgrath**

Hong Kong

**Gerald Mckenna**

Ireland

**Roddy Mcmillan**

UK

**Daniel Mcneil**

USA

**Raghavendra Medikeri**

India

**Lei Mei**

Hong Kong

**Josete Meira**

Brazil

**Jessye Melgarejo Do Amaral Giordani**

Brazil

**Camila Mello Dos Santos**

Brazil

**Theodorus Mettes**

Netherlands

**Hendrik Meyer-Lückel**

Germany

**Edgard Michel-Crosato**

Brazil

**Steffen Mickenautsch**

South Africa

**Peter Milgrom**

USA

**Richard Miron**

Canada

**Haizal Mohd Hussaini**

New Zealand

**Rahim Moineddin**

Canada

**Gustavo Molina**

Argentina

**Andrea Mombelli**

Switzerland

**Fatemeh Momen-Heravi**

USA

**Carlos Moreira**

Brazil

**Susan Morison**

UK

**Manabu Morita**

Japan

**Peter Mossey**

UK

**Sandra Kiss Moura**

Brazil

**Vanessa Muirhead**

UK

**Jan Mulder**

Netherlands

**Andargachew Mulu**

Ethiopia

**Till Mutzbauer**

Switzerland

**Louis Muwazi**

Uganda

**Mangala Nadkarni**

Australia

**Ramesh Nagarajappa**

India

**Rahul Naidu**

Trinidad and Tobago

**Mohammad Zakaria Nassani**

Syria

**Maria Fidela Navarro**

Brazil

**Daniel Nebel**

Sweden

**Klaus Neuhaus**

Switzerland

**Frederico Neves**

Brazil

**Bruce Newbold**

Canada

**Tim Newton**

UK

**Wei Cheong Ngeow**

Malaysia

**Jeff Nickel**

USA

**Dahlia Nielsen**

USA

**Fernando Nogueira**

Brazil

**Fabio Nunes**

Brazil

**Martha Nunn**

USA

**Nazik Nurelhuda**

Sudan

**Karin Nylander**

Swaziland

**Won-Suk Oh**

USA

**Jooyoung Ohe**

South Korea

**Takahiko Oho**

Japan

**Oladapo Olasoji**

Nigeria

**Guilherme Oliveira**

Brazil

**Luciana Butini Oliveira**

Brazil

**Thais Marchini Oliveira**

Brazil

**Lucy O'Malley**

UK

**Edwin Ongkosuwito**

Netherlands

**Olanrewaju Opeodu**

Nigeria

**Omolola Orenuga**

Nigeria

**John Orloff**

Denmark

**Michael O'Rorke**

UK

**Anna-Lena Ostberg**

Sweden

**Janine Owens**

UK

**Elizabeth Oziegbe**

Nigeria

**Damla Özsu**

Turkey

**Nikolaos Pandis**

Switzerland

**Sharat Chandra Pani**

Saudi Arabia

**Bong-Wook Park**

South Korea

**Luis Passeri**

Brazil

**Shankargouda Patil**

India

**Allan Pau**

Malaysia

**Ruben Pauwels**

Belgium

**Adrian Kasaj**

Germany

**Michael Pedersen**

Denmark

**Filiz Namdar Pekiner**

Turkey

**Eduardo Pellizzer**

Brazil

**Sonia Peralta**

Brazil

**Antonio Carlos Pereira**

Brazil

**Karen Peres**

Australia

**Marco Peres**

Australia

**Karin Persson**

Sweden

**Juliano Pessan**

Brazil

**Lestriez Philippe**

France

**Marco Piccinini**

Switzerland

**Ericka Pinheiro**

Brazil

**Teresa Pinho**

Portugal

**Chaiana Piovesan**

Brazil

**Wilson Poi**

Brazil

**Argy Polychronopoulou**

Greece

**Anne Havemose Poulsen**

Denmark

**Peter Proff**

Germany

**Lamis Rajab**

Jordan

**Gordon Ramage**

UK

**Joana Ramos-Jorge**

Brazil

**Cameron Randall**

USA

**Sue Reed**

USA

**Daniel Reissmann**

Germany

**Torsten W. Remmerbach**

Germany

**Morayma Reyes**

USA

**Derek Richards**

UK

**Peter Robinson**

UK

**Rachel Rocha**

Brazil

**Angelo Giuseppe Roncalli**

Brazil

**Irving Rootman**

Canada

**Vinicius Rosa**

Singapore

**Gabriele Rosano**

Italy

**Marina Roscoe**

Brazil

**Charles Rwenyonyi**

Uganda

**Atsushi Saito**

Japan

**Christian Salazar**

USA

**Luciana Sanglard**

Brazil

**Fabio Santos**

Brazil

**Alan Roger Santos-Silva**

Brazil

**Luciana Saraiva**

Brazil

**Gargi Sarode**

India

**Takuichi Sato**

Japan

**Rob Mh Schaub**

Netherlands

**Ulrich Schlagenhauf**

Germany

**Andrea Maria Schmidt-Westhausen**

Germany

**Joel Schwartz**

USA

**Eli Schwarz**

USA

**Falk Schwendicke**

Germany

**Suzanne Scott**

UK

**Parish Sedghizadeh**

USA

**Chaminda Jayampath Seneviratne**

Singapore

**Benedict Seo**

New Zealand

**Patrick Sequeira-Byron**

Switzerland

**Harold Sgan-Cohen**

Israel

**Siddharth Shanbhag**

India

**Aubrey Sheiham**

UK

**Reiko Shibazaki-Yorozuya**

Japan

**Junko Shimomura-Kuroki**

Japan

**Leonie Short**

Australia

**Dmitry Shungin**

Sweden

**Lea Silva**

Brazil

**Sónia Silva**

Portugal

**Carla Sipert**

Brazil

**Anna Skalniak**

Poland

**Per Sodersten**

Sweden

**Evelyne Soriano**

Brazil

**Timo Sorsa**

Finland

**Gianrico Spagnuolo**

Italy

**Giuseppe Spano**

Italy

**Joao Paulo Steffens**

Brazil

**Joanna Stewart New**

Zealand

**Robert Stewart**

UK

**Stefan-Ioan Stratul**

Romania

**Priya Subramaniam**

India

**Karin Sunnegårdh-Grönberg**

Sweden

**Elisabeth Svensson**

Sweden

**Nadia Taher**

Saudi Arabia

**Masato Tamura**

Japan

**Haiping Tan**

Australia

**Keiko Tanaka**

Japan

**Mario Tanomaru-Filho**

Brazil

**Tamara Tedesco**

Brazil

**Britta Tendal**

Denmark

**Naichia Teng**

Taiwan

**Dana Teusner**

Australia

**Leticia Theodoro**

Brazil

**Erika Thomaz**

Brazil

**Brandy Thompson**

Canada

**William Murray Thomson**

New Zealand

**Svetlana Tikhonova**

Canada

**Mon Mon Tin-Oo**

Malaysia

**Scott Tomar**

USA

**Hüseyin Sinan Topçuoglu**

Turkey

**Bálint Török**

Hungary

**Asterios Triantafyllou**

UK

**Simone Tuchtenhagen**

Brazil

**Koichiro Ueki**

Japan

**Makoto Umeda**

Japan

**Aslihan Usumez**

Turkey

**Gerry Uswak**

Canada

**Sabeel P Valappil**

UK

**Ana Maria Valença**

Brazil

**Sigrid Van Den Branden**

Belgium

**Andrea Maria Vargas**

Brazil

**Fabiana Vargas-Ferreira**

Brazil

**Mario Vettore**

UK

**Alexandre Rezende Vieira**

USA

**Jorma Virtanen**

Finland

**Madhu Wagle**

Norway

**Viveca Wallin Bengtsson**

Sweden

**Kristina Wanyonyi**

UK

**Saman Warnakulasuriya**

UK

**Rebecca Wassall**

UK

**Florian J. Wegehaupt**

Switzerland

**Eeva Widström**

Finland

**Annette Wiegand**

Germany

**Leslie Will**

USA

**Raymond Wong**

Singapore

**Sarah Wong Hong**

Kong

**Noel Woods**

Ireland

**Devina Worsley**

UK

**Helen Worthington**

UK

**Samuel Xavier**

Brazil

**Xin Xu**

China

**Jaya Parkash Yadav**

India

